# Prospective cohort study on the social determinants of health: Tehran University of Medical Sciences employees` cohort (TEC) study protocol

**DOI:** 10.1186/s12889-020-09798-9

**Published:** 2020-11-13

**Authors:** Saharnaz Nedjat, Ramin Mehrdad, Masud Yunesian, Hamidreza Pouragha, Vali Biagi, Mohammad Reza Monazzam-Esmaeelpour

**Affiliations:** 1 Department of Epidemiology and Biostatistics, School of Public Health, Knowledge Utilization Research Center, Tehran University of Medical Sciences, Tehran, Iran; 2 Center for Research on Occupational Diseases, Tehran University of Medical Sciences, Tehran, Iran; 3 Department of Environmental Health Engineering, School of Public Health, Department of Research Methodology and Data Analysis, Institute for Environmental Research, Tehran University of Medical Sciences, Tehran, Iran; 4 Department of Occupational Health, School of Public Health, Tehran University of Medical Sciences, Tehran, Iran; 5 Sina Trauma and Surgery Research Center, Tehran University of Medical Sciences, Tehran, Iran

**Keywords:** Social determinant of health, Occupational health, Cohort, Protocol

## Abstract

**Background:**

In this study, the association between the social determinants of health (SDH) as well as other health risk factors and outcomes will be evaluated at different socioeconomic layers.

**Methods/design:**

This is a prospective cohort study that was launched in January 2018 on Tehran University of Medical Sciences’ employees. The initial enrolment phase will continue up to March 2021, or until a sample size of 5500 is reached. In addition to annual phone-calls, the participants will be followed thrice at 5-year intervals. Data are collected through blood and urine samples, complete physical examination, anthropometric evaluation, and the completion of questionnaires related to SDH, such as socioeconomic status and social capital, history of diseases, lifestyle (including, nutrition, physical activity, cigarette and hookah smoking), occupational exposures (including psychosocial factors at work and work-family conflicts), and different aspects of physical, mental and occupational health as health outcomes. The association between independent variables and health (objective or subjective) are examined using multiple models and by controlling the confounding effects. Moreover, the trend in lifestyle changes and its impact on health are evaluated.

**Discussion:**

Our study will explore the key social determinants as well as other factors including socioeconomic status and social capital, history of diseases, lifestyle and occupational exposures that affect health. This will provide social and occupational health decision-makers and stakeholders with new and valuable evidence in an era in which we are witnessing huge changes in lifestyle.

**Supplementary Information:**

The online version contains supplementary material available at 10.1186/s12889-020-09798-9.

## Background

The evaluation of health and its determinant factors in a prospective longitudinal study among a population can yield valuable evidence for the assessment of incidence of various diseases and their risk factors for decision-making. There is an abundance of valid evidence on the risk factors of communicable and most non-communicable diseases, some of which are the result of prospective longitudinal researches. Although the association between social determinants and health outcomes has been studied in several cohort and longitudinal studies [1–4], to the best of our knowledge, we did not find a cohort study in which all the important socioeconomic indicators [5] (education, income, occupation, subjective socioeconomic status, wealth index, childhood socioeconomic status, social capital, and social activities) alongside other factors (as covariates or confounders) have been comprehensively measured in association with various health outcomes. In our study, in comparison to other national and international cohort studies, more detailed social determinants of health (SDH) and economic indicators have been measured [1–4, 6]. In addition, other variables measured as covariates or confounders are more comprehensively compared to other studies. Some SDH, such as education and income, may not necessarily depend on culture; however, some of these factors, such as social capital and social activities are culture-dependent and therefore are not generalizable to other societies [7]. The impact of socio-economic inequalities on health may be culture-dependent [8, 9]. Moreover, there are many interactions between culture and SDH in their associations with health outcomes.

Furthermore, in spite of the recognition of many occupational risk factors, occupational health is facing new challenges due to changes in jobs and lifestyle, the work–family conflict, and the emergence of new occupational psychosocial risk factors. The degree of risk of certain occupational exposures is still unknown, and the mutual impacts of occupational risk factors on other SDH are also unknown. Many of these factors, be it at a less severe rate, are observed in non-employed populations as well.

Tehran University of Medical Sciences (TUMS) is the first established and largest institute of medical sciences in Iran with approximately 19,000 employees [10]. The variety of the employees’ socioeconomic status (SES), easy access to this group, as well as psychosocial similarities in some cases alongside the variety of different occupational levels’ SES, creates a good opportunity to evaluate the incidence of diseases and social risk factors in generalizable form at different layers of the population.

Here, physical, mental, social, environmental and occupational health outcomes are evaluated alongside self-rated health. Occupational diseases are caused by exposure to hazards at the workplace, including physical, chemical, ergonomic, biological, and psychological factors, most of which may be investigated among TUMS employees. Almost all these diseases are completely preventable at the workplace. Given that the adjusted association between social and occupational factors and health outcomes can be investigated upon measuring all the health risk factors, this study measures the other health risk factors as well. Thus, in addition to social and occupational factors, it is possible to examine the association of non-social determinants of health, too. Lifestyle changes such as addiction to smartphones or social networks, or novel types of tobacco have given rise to new risk factors, on the health complications of which there is inadequate evidence. Therefore, the results of this study can provide social and occupational health decision-makers and stakeholders with new and valuable evidence in an era in which we are witnessing tremendous changes in lifestyle.

## Methods and design

### Study design

This is a prospective cohort study. At least three follow-ups will be done at 5-year intervals.

### Objectives

Here, we examine the association between different variables such as socioeconomic status (SES), social capital, job category, occupational risk factors and various health outcomes, namely physical, mental, social, environmental and occupational. Furthermore, the trends in lifestyle changes, health risk factors and the incidence of health outcomes will be investigated. All these investigations are done upon adjustment of other known biological factors such as biochemical and bodily measurements and environmental exposures.

The specific questions being addressed in this research are:

1. Upon controlling the confounding effect of other life risk factors in different occupational groups, are the social determinants of health associated with different objective and subjective health outcomes?
2. What impact do occupational psychosocial risk factors have on the health of different occupational groups after controlling the other health risk factors?
3. How are the trends in changes of lifestyle, work–family conflict, and social capital among different occupational groups and in comparison with each other? How are they associated with various health outcomes?

### Study site

Conveniently accessible from all points of Tehran, with an area of 350 m^2^, the TUMS cohort center is located at the center of the city, and is 5-min walking distance from the main university campus. Furthermore, being located in the comprehensive research laboratory department of the university, it is equipped with advanced laboratory and biobank maintenance equipment. It is also equipped with several rooms intended for interviews, clinical examinations, spirometry, electrocardiography, anthropometry, oral and dental examinations, optometry and audiometry.

### Population under study

Any kind of employment status within TUMS and its affiliated centers, and consent to participate in the study are the inclusion criteria. There are no exclusion criteria.

### Sample size

In cohort studies, different methods are applied to calculate the sample size. Usually, in these studies, the researchers’ goal is to measure multiple exposures and outcomes. Although in recent years many big cohort studies have not fully explained the approach taken to calculate their sample sizes, they have indicated the specific sample size and power needed to statistically demonstrate minimum significant effects (such as odds ratio, etc.) [11, 12]. Applying the same approach, five thousand participants are enough to find 25% difference between two groups with 20% exposure prevalence, with a power of 90% and type 1 error of 5%. Considering 10% loss to follow-up the necessary sample size is 5500.

### Enrolment phase

The first phase of this study was the enrolment phase that began in January 2018 and will continue until March 2021 or until the sample size reaches 5500 (when either goal is achieved the enrolment phase will end).

To inform the University personnel, official invitations were made by the university’s deputy of research and the human resources management to all the faculties and centers affiliated with the university. Moreover, all centers were asked to introduce one person as a focal point to introduce their volunteers to the cohort center. The study’s poster and brochure were also sent to TUMS’ centers to present the cohort’s goals and executive phases to the participants. Enrolment is done via phone, short message service (SMS), the study’s website, through the focal points, and in person. Group enrolment is performed by sending official letters on behalf of the university to different TUMS’ centers. The hours the personnel attend the cohort center are considered as daily assignments. When making appointments, participants are asked to fast for a period of 12 h before arriving at the cohort center. The participants are asked to give their written consent upon their first visit.

### Variables under study

The list of variables and items measured at the baseline phase of this study are summarized in Additional file 1.

#### Laboratory tests

During the first stage, biological samples (25 ml blood and 20 ml urine) are collected from each participant and the following tests are done: fasting blood sugar, blood urea, creatinine, uric acid, total cholesterol, Triglyceride, High-density lipoprotein (HDL) cholesterol, low-density lipoproteins (LDL) cholesterol, Aspartate Aminotransferase (AST), Alanine Aminotransferase (ALT), Alkaline Phosphatase (ALP), complete blood cell count (CBC) and Urine analysis (UA). Once blood and urine samples are taken, breakfast is served. For each individual, 10 blood samples are kept at − 80 degrees Celsius (including whole blood, plasma, serum, Buffy coat and DNA), and two urine samples are kept at − 30 degrees Celsius in the biobank. The necessary analyses (mostly nested) will be conducted on these samples during the follow-ups. The preliminary lab results are stamped by the lab technician and handed over to the participant in printed form on the same day before leaving the cohort center. Another copy interpreted by the physician is handed over to the cohort study at the same time.

In addition to the blood and urine tests, data on the independent variables and outcomes of the participants are collected through two methods:

a- *Questioning by trained interviewers and completion of questionnaires*, the validity and reliability of which have been previously approved among Iran’s population. The Socioeconomic Status of participants is evaluated by different indicators. These indicators include asset – based measures (wealth index), educational status, occupation, income, insurance coverage and subjective socioeconomic status (SSS), as well as other social variables like social capital and social activities including frequency of going to concerts, the theater or cinema [13].

#### Asset-based socioeconomic indicator (wealth index)

The asset questions included possession of privately owned cars (its price is assessed), motorcycles, smartphones, freezers, dishwashers, microwave ovens, personal computers, vacuum cleaners, washing machines, internet access at home, color TVs, DVD players, the frequency of dining at restaurants at personal cost, trips by plane and monthly internet costs. The living space per capita and the number of rooms per capita are also questioned. The categorical principal component analysis (CATPCA) will be applied to the net assets of each participant and the wealth index will be created [13].

#### Educational status

Educational status is measured in terms of years of formal education completed successfully and the qualifications attained.

#### Subjective socioeconomic status (SSS)

SSS is measured with the following question: “what would your household position be if the socioeconomic status of your community was divided into the five categories?” [14, 15].

#### Occupation

For all participants, the job title, employment status, and the occupational groups (including office and administrative services, clinical and medical services, public services, technical services, diagnostic and chemical laboratory staff, and security guards) are assessed. Further participant data are collected with regards to the employment status, namely the job category, management level, professional records, shift work, type of insurance and spouse’s job.

#### Childhood socioeconomic status

To measure the childhood SES, the following question is included: *“what would your position be if the socioeconomic status of your community when you were 18 years old was divided into the five categories?”* The patients are asked to answer the abovementioned question by choosing one of the following *“Low, Moderate to low, Moderate, Moderate to high, and High”.* Also, parental educational status and fluctuations of the household socioeconomic status over time are measured [16].

Social capital questions have been derived from the World Bank questionnaire for developing countries, which has demonstrated good reliability and validity in Iran [17]. In addition, we will be assessing macro-social determinants of health including income inequality, income growth, unemployment rates, national income, migration, taxation policies and the country’s international policies [18, 19].

Questions related to lifestyle include International Physical Activity Questionnaire-Short Form (IPAQ-SF) [20], exposure to cigarettes, being a passive smoker and hookah smoker [21], the dish-based semi-quantitative food frequency questionnaire (FFQ) [22], oral health, sleep quality and quantity. Sleep quantity and quality are evaluated with the Insomnia Severity Index [23].

Since alcohol consumption is forbidden by law and jurisprudence in Iran and this study has been conducted in TUMS as the participants’ workplace, large under-reporting is expected, therefore, we do not ask about it. Quality of life is measured using the World Health Organization Quality of Life Brief questionnaire (WHOQOL- BREF) [24].

Other questionnaires that are used to collect data are questionnaires on workplace – related exposures and outcomes. These include the Work–Family Conflict Scale [25], the Copenhagen Psychosocial Questionnaire (COPSOQ) on the psychosocial conditions of the workplace [26], the Kessler Psychological Distress Scale (K10) [27], and the questionnaire that assesses Depression Anxiety, and Stress (DASS) in the population under study [28]. Aggression, suicidal thoughts and attempts, and resilience are determined by the questionnaire for assessment of aggression [29], questionnaire for suicidal thoughts and behaviors [30] and the Connor-Davidson Resilience Scale (CD-RISD) questionnaires [31, 32].

The smartphone addiction status is assessed with the smartphone addiction scale (SAS). This questionnaire has been translated and validated in Iran. The Persian version of SAS had an acceptable reliability and validity for assessing smartphone addiction. The intra-class correlation coefficient of the questionnaire was 0.86. Questions are asked regarding history of diseases in 17 groups and an overall number of 139 diseases which are listed in the experts’ sessions. Also, surgical records are examined in 9 separate groups. Moreover, questions on current diseases, medications used, reproductive health, history and behavior have also been designed and inserted.

The ROSE questionnaire is used to examine the cardiac health status and history of chest pain [33]. The STOP-Bang sleep apnea [34] questionnaire and the Dyspnea Checklist [35] are also completed. All the questionnaires are completed through the interviews, except for the FFQ, which is self-administered –once the nutritionist gives the necessary explanations to the participants.

b- *Medical Examination*

The general examination includes cardiovascular examination and systolic and diastolic blood pressure measurement by a general physician. Oral and dental hygiene is examined by a dentist –using the relevant checklist and the WHO guide [36]. The following tests/examinations are conducted by trained and experienced experts with specialties in nursing, optometry, audiology, nutrition, and physiotherapy: body size and composition, spirometry, audiometry (air and bone conduction threshold and speech tests), optometry (refraction and autorefractometry, visual acuity determination by the LED chart, lensometry and retinoscopy), Electrocardiography (ECG), dynamometry of hands and fingers by the Tip pinch, lateral pinch, key pinch, squeezing and hand grip tests.

#### Environmental quality metrics

The environmental factors will be evaluated via two strategies: Firstly, we will measure the most important chemical pollutants in the bio-specimen that has been taken from the participants and bio-banked at minus 72 degrees Celsius. These pollutants include heavy metals, persistent organic pollutants (POPs), pesticides and endocrine-disrupting compounds (EDCs), and other pollutants by indication and will be measured in the serum, plasma, or urine samples of the participants in a nested case-control fashion. Secondly, criteria air pollutants (PMs, NOx, SOx, Ozone, CO, and Lead) measured in more than 20 monitoring stations thought Tehran great metropolitan area will be allocated based on the residence and work location of participants using GIS measures. These pollutants will be calculated and assigned to each participant in both short term (maximum concentration in the period of study) and long term (averaged concentration in the different periods of time based on the hypotheses of the study) scenarios.

### Determining the study’s validity

All the interviewers and examiners were trained and began their work after quality assurance. Throughout the study, the data are controlled on a daily basis and in case any issues are detected, the quality control manager refers the issue to the relevant expert by completing a form. Moreover, the reasons behind the lack of coordination will be inspected in the presence of the project executives. Data analysis is conducted once a month to monitor their quality. The research data are saved on a specific server with a high level of security. Backup of the data is saved every 2 weeks and stored in three separate physical and spatial caches.

### Follow-up phase

Follow-up will be done every 5 years. The exposures and outcomes will be re-examined. The participants will be annually contacted via phone and any changes in the important outcomes will be asked.

In this study, different approaches are used to reduce the dropout rate. The approaches for preventing dropout include registration of contact information on the participants, their friends and relatives (postal address, home phone number, work phone number, cellphone number, and email address), ensuring good communication between the cohort staff and participants (using well trained and enthusiastic staff and open communication), easy accessibility to the cohort center (the TUMS cohort center is located at the center of the city, and is 5-min walking distance from the main university campus), effective communication channels, regular and rapid feedback on the participant’s health information, regular contacts with the participants (annual phone interviews with all the participants to collect data on key variables and outcomes). Finally, since we will need to address the degree to which dropout might bias those results, we document the reasons the participants leave the study; we also note down the participants’ characteristics.

### Ethical considerations

This study acquired two ethical registration codes, as it consisted of two phases, the pilot study and the main study. They have been registered by TUMS ethics committee and the Ministry of Health and Medical Education under registration codes IR.TUMS.VCR.REC.1396.4265 and IR.TUMS.VCR.REC.1398.246, respectively. Before registration, all participants read and sign the written consent form. A copy of the signed consent form is given to the participant. The consent form includes the use of biobank samples for future studies. All data are kept confidential and confidential codes are required to open them, which is only possible by the project executive. The participants are informed of their health status by the physician on the same day, and referred to relevant specialists if immediate health interventions are necessary.

### Statistical analysis

Initially, descriptive statistics will be used to describe the baseline characteristics of the participants. Normally distributed variables will be described by the mean and standard deviation. In the case of non-normal distributions, the data will be presented as median and interquartile range (IQR). Also, frequency and percent will be calculated for qualitative variables. The incidence of different health outcomes will be determined in the exposed and non-exposed groups. We will assess whether there are significant differences between health risk factors and outcomes by means of different regression models. The type of regression will be based on the type of outcome variables. Time-dependent models and survival models will be applied for the analysis of follow-up and repeated measures data. All regression models will be adjusted for potential confounder variables. The concentration index and the relative index of inequality (RII) will be used to measure socioeconomic inequality in health. *P* ≤ 0.05 will be considered as statistically significant.

## Discussion

This study will explore the key social determinants as well as other factors including socioeconomic status and social capital, history of diseases, lifestyle and occupational exposures that affect health. This will provide social and occupational health decision-makers and stakeholders with new and valuable evidence in an era in which we are witnessing tremendous changes in lifestyle.

The results of this study will be published in well-known international journals. National and international dissertations based on the project’s data will be welcomed on condition of referral to the cohort, in accordance with its documented guidelines (Additional file 2). The research results will be presented to the university’s managers and national decision-makers in oral and written form.

## Supplementary Information

**Additional file 1:** **Table S1.** Domains and items covered in the data collection of the first phase.

**Additional file 2.** Executive directive on the manner of cooperation with Tehran University of Medical Sciences (TUMS) employees` cohort study (TEC) for publication of the results.

## Data Availability

Not Applicable. However, the datasets collected during the current study are available from the corresponding author on reasonable request (Additional file 2).

## Abbreviations

SDH: Social Determinants of Health
TUMS: Tehran University of Medical Sciences
SES: Socioeconomic Status
SSS: Subjective Socioeconomic Status
COPSOQ: Copenhagen Psychosocial Questionnaire
K10: Kessler Psychological Distress Scale
DASS: Depression. Anxiety and Stress Scale
CD-RISD: Connor-Davidson Resilience Scale
SAS: Smartphone Addiction Scale
HDL: High-density lipoprotein (HDL)
LDL: Low-Density Lipoproteins
AST: Aspartate Aminotransferase
ALT: Alanine Aminotransferase
ALP: Alkaline Phosphatase
CBC: Complete Blood Cell Count
UA: Urine analysis
SMS: short message service
IPAQ-SF: International Physical Activity Questionnaire-Short Form
FFQ: Food Frequency Questionnaire
QOL-SF: Quality of Life Questionnaire Short Form
ECG: Electrocardiography

## References

[1] Marmot M, Brunner E (2005). Cohort profile: the Whitehall II study. Int J Epidemiol.

[2] Steptoe A, Breeze E, Banks J, Nazroo J (2013). Cohort profile: the English longitudinal study of ageing. Int J Epidemiol.

[3] Fotouhi A, Hashemi H, Shariati M, Emamian MH, Yazdani K, Jafarzadehpur E, Koohian H, Khademi MR, Hodjatjalali K, Kheirkhah A (2013). Cohort profile: Shahroud eye cohort study. Int J Epidemiol.

[4] Pasdar Y, Najafi F, Moradinazar M, Shakiba E, Karim H, Hamzeh B, Nelson M, Dobson A: Cohort profile: Ravansar Non-Communicable Disease cohort study: the first cohort study in a Kurdish population. Int J Epidemiol 2019, 48(3):682- 683f. 10.1093/ije/dyy296 30753701

[5] Braveman P, Gottlieb L: The social determinants of health: it's time to consider the causes of the causes. Public Health Reports 2014, 129(1_suppl2):19–31. 10.1177/00333549141291S206 PMC3863696 24385661

[6] Pourshams A, Khademi H, Malekshah AF, Islami F, Nouraei M, Sadjadi AR, Jafari E, Rakhshani N, Salahi R, Semnani S (2010). Cohort profile: the Golestan cohort study—a prospective study of oesophageal cancer in northern Iran. Int J Epidemiol.

[7] Solar O, Irwin A (2010). A conceptual framework for action on the social determinants of health. WHO Document Production Services.

[8] Sweeting H, West P (1995). Family life and health in adolescence: a role for culture in the health inequalities debate. Soc Sci Med.

[9] Ryff CD, Miyamoto Y, Boylan JM, Coe CL, Karasawa M, Kawakami N, Kan C, Love GD, Levine C, Markus HR (2015). Culture, inequality, and health: evidence from the MIDUS and MIDJA comparison. Culture Brain.

[10] Tehran University of Medical Sciences. Statistics and Information Technology Management. 2019. http://sit1.tums.ac.ir/index.jsp?siteid=2&fkeyid=&siteid=2&pageid=11304. Accessed 15 Dec 2019.

[11] Golding J, Steer C (2009). How many subjects are needed in a longitudinal birth cohort study?. Paediatr Perinat Epidemiol.

[12] Brown RC, Dwyer T, Kasten C, Krotoski D, Li Z, Linet MS, Olsen J, Scheidt P, Winn DM (2007). Cohort profile: the international childhood cancer cohort consortium (I4C). Int J Epidemiol.

[13] Sartipi M, Nedjat S, Mansournia MA, Baigi V, Fotouhi A: Assets as a socioeconomic status index: categorical principal components analysis vs. latent class analysis. Archives Iran Med 2016, 19(11):0–0. 27845549

[14] Baigi V, Nedjat S, Fotouhi A, Janani L, Mohammad K (2016). Subjective social status in association with various health and socioeconomic indicators in Tehran. J Public Health.

[15] Singh-Manoux A, Adler NE, Marmot MG (2003). Subjective social status: its determinants and its association with measures of ill-health in the Whitehall II study. Soc Sci Med.

[16] Kittleson MM, Meoni LA, Wang N-Y, Chu AY, Ford DE, Klag MJ (2006). Association of childhood socioeconomic status with subsequent coronary heart disease in physicians. Arch Intern Med.

[17] Yari A, Nadrian H, Rashidian H, Nedjat S, Esmaeilnasab N, Doroudi R, Hoursan H (2014). Psychometric properties of the Persian version of social capital questionnaire in Iran. Med J Islam Repub Iran.

[18] Muntaner C, Chung H, Solar O, Santana V, Castedo A, Benach J, Network E (2010). A macro-level model of employment relations and health inequalities. Int J Health Serv.

[19] Muntaner C, Chung H (2008). Commentary: macrosocial determinants, epidemiology, and health policy: should politics and economics be banned from social determinants of health research?. J Public Health Policy.

[20] Vasheghani-Farahani A, Tahmasbi M, Asheri H, Ashraf H, Nedjat S, Kordi R (2011). The Persian, last 7-day, long form of the international physical activity questionnaire: translation and validation study. Asian J Sports Med.

[21] Hessami Z, Masjedi MR, Mortaz E, Heydari G, Kazempour-Dizaji M, Sharifi H, Jamaati H (2016). Evaluation of dual tobacco smoking (water pipe and cigarettes) and associated factors in adults in Tehran. Tanaffos.

[22] Keshteli AH, Esmaillzadeh A, Rajaie S, Askari G, Feinle-Bisset C, Adibi P (2014). A dish-based semi-quantitative food frequency questionnaire for assessment of dietary intakes in epidemiologic studies in Iran: design and development. Int J Prev Med.

[23] Yazdi Z, Sadeghniiat-Haghighi K, Zohal MA, Elmizadeh K (2012). Validity and reliability of the Iranian version of the insomnia severity index. Malaysian J Med Sci.

[24] Nedjat S, Montazeri A, Holakouie K, Mohammad K, Majdzadeh R (2008). Psychometric properties of the Iranian interview-administered version of the World Health Organization's quality of life questionnaire (WHOQOL-BREF): a population-based study. BMC Health Serv Res.

[25] Mozafari M, Azami G, Dehkordi ML, Aazami S (2016). Validation of multidimensional Persian version of the work-family conflict questionnaire among nurses. Int J Occupational Environ Med.

[26] Pournik O, Ghalichi L, TehraniYazdi A, Tabatabaee SM, Ghaffari M, Vingard E (2015). Measuring psychosocial exposures: validation of the Persian of the Copenhagen psychosocial questionnaire (COPSOQ). Med J Islam Repub Iran.

[27] Hajebi A, Motevalian A, Amin-Esmaeili M, Rahimi-Movaghar A, Sharifi V, Hoseini L, Shadloo B, Mojtabai R (2018). Adaptation and validation of short scales for assessment of psychological distress in I ran: the P ersian K10 and K6. Int J Methods Psychiatr Res.

[28] Asghari A, Saed F, Dibajnia P (2008). Psychometric properties of the depression anxiety stress Scales-21 (DASS-21) in a non-clinical Iranian sample. Int J Psychol.

[29] Mojtabai R (2006). Psychotic-like experiences and interpersonal violence in the general population. Soc Psychiatry Psychiatr Epidemiol.

[30] Borges G, Nock MK, Abad JMH, Hwang I, Sampson NA, Alonso J, Andrade LH, Angermeyer MC, Beautrais A, Bromet E (2010). Twelve month prevalence of and risk factors for suicide attempts in the WHO world mental health surveys. J Clin Psychiatry.

[31] Connor KM, Davidson JR (2003). Development of a new resilience scale: the Connor-Davidson resilience scale (CD-RISC). Depression Anxiety.

[32] Rahimi-Movaghar A, Amin-Esmaeili M, Sharifi V, Hajebi A, Radgoodarzi R, Hefazi M, Motevalian A (2014). Iranian mental health survey: design and field proced. Iran J Psychiatry.

[33] Rose GA (1962). The diagnosis of ischaemic heart pain and intermittent claudication in field surveys. Bull World Health Organ.

[34] Sadeghniiat-Haghighi K, Montazeri A, Khajeh-Mehrizi A, Ghajarzadeh M, Alemohammad ZB, Aminian O, Sedaghat M (2015). The STOP-BANG questionnaire: reliability and validity of the Persian version in sleep clinic population. Qual Life Res.

[35] Nurses AAoHF (2020). Dyspnea checklist.

[36] Organization WH (2013). Oral health surveys: basic methods: World Health Organization.

