# Supplementary material for: Prospective cohort study on the social determinants of health: Tehran University of Medical Sciences employees` cohort (TEC) study protocol
Source: BMC Public Health. 2020 Nov 13;20:1703. doi: 10.1186/s12889-020-09798-9 (PMC7666496; doi:10.1186/s12889-020-09798-9)
Supplement: Supplementary file 1 — Additional file 1: Table S1. Domains and items covered in the data collection of the first phase. [file 12889_2020_9798_MOESM1_ESM.docx]

| Table S1: Domains and items covered in the data collection of the first phase | |
| --- | --- |
| Socioeconomic factors | Asset-based socioeconomic indicator (wealth index)  Income  Occupation (job category, employment history, employment status, management level, professional records, shift work)  Subjective socioeconomic status (SSS)  Childhood socioeconomic status  Spouse’s job  Education  Parent`s education  Social capital  Social activities  Ethnicity  Tendency of migration  Type of insurance  Fluctuations in socioeconomic |
| Quality of life and lifestyle | Quality of life  Physical Activity  Cigarette  Being a passive smoker  Hookah smoke  Oral Health  Food frequency  Sleep quality |
| Mental and Spiritual health | Smartphone addiction  Stress  Anxiety  Depression  Psychological Distress  Aggression  Suicidal thoughts and attempts  Resilience  Work-family conflict |
| Reproductive history | Number of births  Live births  Miscarriages  Stillbirths  History of breastfeeding  Age at menarche/menopause  Age at menopause  History of contraception  Contraception method  History of hysterectomy  Tube ligation  History of infertility  History of breast screening  History of cervical cancer screening |

| Table S: continued…. | |
| --- | --- |
| Laboratory tests | Fasting blood sugar  Blood urea  Creatinine  Uric acid  Total cholesterol  Triglyceride  High-density  Lipoprotein (HDL) cholesterol  Low-density lipoproteins (LDL) cholesterol  Aspartate Aminotransferase (AST)  Alanine Aminotransferase (ALT)  Alkaline Phosphatase (ALP)  Complete blood cell count (CBC)  Urine analysis (UA) |
| General and medical Examination | Blood pressure (diastolic and systolic)  Pulse rate  Hip circumference  Waist circumference  Height  Weight  Full body composition  Mid-upper arm circumference  Thigh circumference  Hand dynamometer  Electrocardiogram  Audiometry  Optometric examinations  Spirometry  Self-rated health |
| Medications (Use of prescribed drugs and supplements) | There are 1320 drugs in the study’s software, based on Iran’s Social Security Organization’s list, which will be registered if used by the participants. |
| Ecological and macrosocial factors | Income inequality  Economic growth  Unemployment rates  National income  Migration  Taxation policies  International policy of the country |
