# Supplementary material for: Prospective cohort study on the social determinants of health: Tehran University of Medical Sciences employees` cohort (TEC) study protocol
Source: BMC Public Health. 2020 Nov 13;20:1703. doi: 10.1186/s12889-020-09798-9 (PMC7666496; doi:10.1186/s12889-020-09798-9)
Supplement: Supplementary file 2 — Additional file 2. Executive directive on the manner of cooperation with Tehran University of Medical Sciences (TUMS) employees` cohort study (TEC) for publication of the results. [file 12889_2020_9798_MOESM2_ESM.docx]

**Executive directive on the manner of cooperation with Tehran University of Medical Sciences (TUMS) employees` cohort study (TEC) for publication of the results**

**Introduction:**

Observance of ethics in research is one of the most important points that is considered in medical researches and practicing these ethics is one of the professional behavior criteria. Observance of these ethical principles in various stages of research such as plan, implementation, data collection and publication of the results is of great importance. Due to the emphasis of Tehran University of Medical Sciences Employees Cohort Study (TUMS Employees Cohort TEC) on observing the ethical principles in all the executive stages of the study, the following directive for publishing the cohort results was passed on -----, by the ----- meeting of Study Steering Committee and it shall be effective as of the date of approval.

**Values:**

Since the TEC study is implemented and financially supported with the general costs and credits of the country, all the scholars and researchers including faculty members of universities and higher education institutes and students of different academic levels inside and outside the country can use the study data by observing the provisions of this directive and can participate in publishing the results of the study.

TEC is committed to observe the authorship ethical principles in compliance with the COPE Protocol and the guideline of International Committee of Medical Journal Editors (ICMJE) and does its best to ensure that authorship criteria are properly observed in all the published papers and articles and that Guest and Ghost Author cases are avoided.

Based on the recommendation of ICMJE, the prerequisite for creating an authorship right is having the following 4 criteria at the same time:

- Active participation in planning the study content or data collection, or analysis and interpretation of the results
- Writing the paper and/or reviewing the paper in terms of scientific contents
- Confirming the final version of the paper
- Responding to the questions related to the various contexts presented in the paper

Therefore,

The TUMS Employees` Cohort Study officially recognizes as authors those who have met the above-mentioned four criteria.

Any of the plan colleagues (including executive, person in charge of the plan, members of Steering Committee, director and/or executive directors, plan colleagues and experts) can benefit from the paper authorship privileges in case of meeting the above criteria for each paper. TEC colleagues, like other researchers, if intend to benefit from the results and participate in publishing the results, are required to observe the steps stated in this directive and need to obtain the necessary license for publishing the papers.

In order to protect the TUMS rights regarding the results published from TUMS Employees Cohort Study, it is necessary to state the following phrases in Acknowledgement section of the papers published in Persian and English.

1. Persian Paper:
2. Thesis :

This paper is the result (a part) of a thesis titled ------------------------------, in the course (Bachelor / Master/ Medical Doctorate / Specialized Doctorate) in year ---- under code ----, which is implemented with the support of TUMS and participation /support of TUMS Employees Cohort Study, under code 36600.

If the thesis is not supported by TUMS:

This paper is the result (a part) of a thesis titled ------------------------------, in the course (Bachelor / Master/ Medical Doctorate / Specialized Doctorate) in year ---- under code ----, which is implemented with the participation /support of TUMS Employees Cohort Study, under code 36600.

1. Plan:

This paper is the result (a part) of a research plan titled ------------------------------, approved by TUMS in year ---- under code ----, which is implemented with the support of TUMS and participation /support of TUMS Employees Cohort Study, under code 36600.

If the plan is not supported by TUMS:

This paper is the result (a part) of a research plan titled ------------------------------, approved by TUMS in year ---- under code ----, which is implemented with the participation /support of TUMS Employees Cohort Study, under code 36600.

**Executive Method:**

The executive steps for the researchers who wish to use the TEC study data are as follows:

- To submit a request to Study Steering Committee for intending to use the data (this request must contain a research plan proposal including the subject and idea, the person responsible for using the data, the requested variables, the names of the team and colleagues, and the intended time till publication of the results)
- To review the issue in Study Steering Committee meeting and provide a response within a maximum duration of two weeks
- To sign a letter of commitment for research cooperation with the TEC Study
- To provide the approved data

Note: The study data will be provided to the researcher after approval of the plan proposal in a research council and competent ethics committee (including faculty, research center, research institute, university) through the above steps.

Letter of Commitment for Research Cooperation with TUMS Employees Cohort Study

I, ---------, daughter/son of -----, born in ----, holder of birth certificate No. ----, National ID No. -----, residing in -------, Tel: ------, faculty member / student / researcher in the field of ------, at ------- University, have commenced my research titled -----------, by using the data of TUMS Employees Cohort (TEC) and commit to precisely implement the ethical principles and intellectual property rights in accordance with the provisions of the Executive Directive on the Manner of Cooperation with TUMS Employee’s Cohort Study (TEC) for Publication of the Results and state the name of TEC center, as per the Executive Directive, in all the published papers in Acknowledgement section and I am not authorized to transfer the data to any researcher /research team without permission of the TEC Study Steering Committee.

In case I fail to submit the paper to a reliable journal up to ----- months after receiving the data and/or fail to obtain the paper acceptance within a maximum duration of one year after submission, TEC Center will be authorized to transfer the data to another person/ research team

Any change in the composition of the research team and/or the title or the project content will occur with knowledge and permission of TEC Center.

Violation of any of the above cases solely is considered as the crime of betrayal of trust and the related authorities will be entitled to pursue the matter legally and claim for the damages incurred to them.

Composition of the research team is as follows:

| Date | Signature | Position in the Study * | Origin Organization | First Name and Surname |
| --- | --- | --- | --- | --- |
|  |  |  |  |  |
|  |  |  |  |  |
|  |  |  |  |  |

* The first author, the person in charge or other cases should be mentioned.
